# The top five research priorities in physician-provided pre-hospital critical care: a consensus report from a European research collaboration

**DOI:** 10.1186/1757-7241-19-57

**Published:** 2011-10-13

**Authors:** Espen Fevang, David Lockey, Julian Thompson, Hans Morten Lossius

**Affiliations:** 1 Department of Research and Development, Norwegian Air Ambulance Foundation, Drøbak, Norway; 2 Department of Anaesthesiology and Intensive Care, Stavanger University Hospital, Stavanger, Norway; 3 London HEMS, The Royal London Hospital, London, UK; 4 School of Clinical Sciences, University of Bristol, Bristol, UK; 5 Department of Surgical Sciences, University of Bergen, Bergen, Norway

## Abstract

**Background:**

Physician-manned emergency medical teams supplement other emergency medical services in some countries. These teams are often selectively deployed to patients who are considered likely to require critical care treatment in the pre-hospital phase. The evidence base for guidelines for pre-hospital triage and immediate medical care is often poor. We used a recognised consensus methodology to define key priority areas for research within the subfield of physician-provided pre-hospital critical care.

**Methods:**

A European expert panel participated in a consensus process based upon a four-stage modified nominal group technique that included a consensus meeting.

**Results:**

The expert panel concluded that the five most important areas for further research in the field of physician-based pre-hospital critical care were the following: Appropriate staffing and training in pre-hospital critical care and the effect on outcomes, advanced airway management in pre-hospital care, definition of time windows for key critical interventions which are indicated in the pre-hospital phase of care, the role of pre-hospital ultrasound and dispatch criteria for pre-hospital critical care services.

**Conclusion:**

A modified nominal group technique was successfully used by a European expert group to reach consensus on the most important research priorities in physician-provided pre-hospital critical care.

## Background

The concept of a physician-manned pre-hospital emergency medical team was born in the early 1950s, and the first physician-manned mobile intensive care unit (MICU) was put into service in Heidelberg, Germany, in 1957 [1]. To expand the area served and reduce transportation times, the first physician-manned *helicopter *emergency medical service (HEMS) became operational in Munich in 1968 [2]. Although ambulance personnel or nurses are usually the first pre-hospital medical personnel to assess the critically ill or injured patient, many countries in Europe and, to some extent, Australasia, commonly deploy physicians, often anaesthesiologists, in pre-hospital emergency medical services (EMS) [3-6]. Physician-staffed EMS are a limited resource due to the capacity and costs associated with the equipment, staffing and training and are often selectively deployed by helicopter or land-based emergency response vehicles to patients considered likely to require critical care treatment in the pre-hospital phase. Dispatch systems differ: some systems utilise immediate call-out criteria based on diagnoses or type of incident, whereas others respond to requests for assistance from non-physician EMS after the initial patient assessment [7-9].

International guidelines for pre-hospital triage and immediate medical care have been developed for several emergency medical conditions. Unfortunately, the evidence base of such guidelines is often poor, particularly in trauma care [10-15]. While numerous studies have analysed the impact of educational interventions, such as Advanced Trauma Life Support™, such data is of limited relevance to pre-hospital care research because the primary focus is on the in-hospital phase of trauma management, and there is a tendency for published studies to assess the educational rather than clinical success of interventions [14,16-20]. More clinically oriented studies usually apply a retrospective approach, for example by comparing trauma deaths before and after the implementation of novel trauma systems. Separation of the pre-hospital component of care from the entire trauma patient pathway limits the utility of these studies and makes reliable conclusions about pre-hospital care difficult [21-24].

The lack of conclusive, reliable and valid research, accompanied by inconsistent and imprecise reporting of data results in little empiric evidence within this field. This may explain the on-going debate regarding key questions in pre-hospital critical care [25-29]. Randomised controlled trials are difficult to conduct given the nature of the field [30-35]. Some authors have even questioned the use of randomised controlled trials in pre-hospital emergency medicine, arguing that the ethical and practical implications may outweigh the potential benefits [36]. This lack of valid, high-quality research is reflected in a relatively small number of systematic reviews compared with areas of in-hospital medical care. Therefore, protocols for pre-hospital critical care are, to a large extent, based on low-level evidence and sometimes little more than expert opinion [10,37].

A British expert group was recently commissioned to review the evidence base for the delivery of emergency pre-hospital care, to identify gaps in the evidence base, and to prioritise topics for future research [38]. This report focuses on non-physician-manned EMSs and non-critical pre-hospital care. Due to the differences in medical competence and the availability of advanced interventions, we believe that a focus on physician-provided care for critically unwell patients is likely to yield different priorities. Confronted by this challenge, we wanted to identify specific areas in need of high-quality research. The aim of our study was to define key priority areas for research within the subfield of physician-provided pre-hospital critical care by using a recognised consensus methodology. We have used the term physician-staffed EMS for all physician-manned pre-hospital emergency medical teams, and this consensus process focused only on those teams that are physician-manned.

## Methods

A panel consisting of European experts in physician-based pre-hospital critical care was invited to participate in the consensus process. The consensus process was based upon a four-stage modified nominal group technique (NGT) that included a consensus meeting [39-42].

### The expert panel

The criteria for selecting representatives for the expert panel were clinical research and guideline development experience in European physician-based pre-hospital critical care. All members of the expert panel were physicians with clinical experience from participation in physician-staffed EMS. We sought to include countries with well-developed and well-integrated physician-staffed EMS similar to the Scandinavian model. The experts were identified through PubMed and Google searches, the professional network of the project group, and proposals from the invited panel members. The experts were invited by e-mail, and three reminders were sent to non-responders.

### The consensus process

The consensus process was conducted in four consecutive stages.

#### Stage one

Each invited expert was asked to use his or her expert opinion and knowledge of relevant research and guidelines to prioritise the five areas in the field of physician-provided pre-hospital critical care that he or she believed were most in need of further research. The explicit aim of the process was to identify important deficits in the current evidence base and to provide a focus for future research. Participants were guided in the scope of their prioritised 'areas' using an example from a separate field of emergency medicine, with 'Imaging in trauma patients' provided as the specimen 'area' in this field. The invited experts were asked to define 'areas' of similar scope in physician-provided pre-hospital critical care. The proposals were returned to the project group on a pre-designed Excel worksheet (Microsoft Office 2008, Microsoft Inc., USA) by e-mail.

#### Stage two

All suggestions from Stage one were categorised and alphabetised by the project group before being presented to the expert panel by e-mail as an Excel file. The participants were asked to consider the entire list of Stage one proposals and to prioritise, in order, their ten most important suggestions. The choice of ten prioritised areas prevented participants from potentially nominating only their own five suggestions from Stage one and allowed a broader foundation on which to base the final results. During Stage two, all participants were encouraged to combine areas they considered to have the same core meaning, implying that each of the ten areas could consist of several suggestions from Stage one.

#### Stage three

In Stage three the prioritised areas from all experts were combined to create an overall list of the priorities. We used a modified version of the system described by Delbecq and Van de Ven in 1971 [43]. Our calculation awarded a point value to each suggestion depending upon its placement in the ranked top ten: a ranking of first place gave a point value of ten, second of nine, third of eight and so forth, until the tenth place conferred one point. In addition, each suggestion was awarded two points each time it was nominated in a participant's top ten list. The total and, hence, the overall importance of each suggested area was calculated from both the ranking priority awarded to it by the expert panel members and by the number of times it was included in the top-ten list of all participants. This process allowed the top five priorities, as suggested by the expert panel, to be identified using simple mathematical equations within the Excel program. A list containing the top five areas and those that finished in sixth to tenth along with comments from the initial stages was returned to the participants two weeks before the consensus meeting. This step enabled all members of the expert panel to perform a second examination of relevant scientific material before the group gathering.

#### Stage four

A consensus meeting was held at Torpo, Norway. This provided the opportunity for extensive discussion and debate in order to reach a final agreement on the top five areas in need of further research. The prioritised areas were further analysed, and three specific and important research questions were identified within each of these five areas. The discussion included suggestions for methodology, practicalities and approximate study size. Subsequent to the consensus meeting, all participants received an e-mailed draft list of the five areas with the attendant three questions in each area. Comments on this draft were taken into consideration before all members of the expert panel agreed on the final table.

## Results

Twenty-one physicians from Europe were invited to join the expert panel, and 18 responded. In addition, another panel member was suggested by one of the invited experts and accepted the invitation, making a total of 19 participating researchers. Five participants were unable to attend the consensus meeting but participated in Stages one, two, three and a review of the consensus meeting results. Fourteen experts were present at the consensus meeting.

A total of 85 different research subject areas were identified after Stage one.

In Stage two, the experts individually combined similar areas of the 85 suggestions, reducing the total number of research areas to 36. (Table 1) In Stage three, the project group applied the prioritisation system described in the methods section and worked out, in a consecutive sequence, the ten areas that received the highest point value. These were: 1: Pre-hospital critical care: Staffing, training and effect, 2: Advanced airway management in pre-hospital care: what is best for the patient? 3: Define time window for time critical interventions - what are the time windows for critical care interventions whether pre- or in-hospital? 4: Pre-hospital ultrasound, 5: Dispatch/activation criteria for pre-hospital critical care services, 6: Integrated information systems, 7: Evaluating quality of care, 8: Patient safety - how to avoid patients getting harmed by pre-hospital procedures and therapies? 9: Pre-hospital temperature management in severely injured and ill patients, 10: Monitoring in the pre-hospital setting.

**Table 1:** All suggested research areas

1	Pre-hospital critical care. Staffing, training and effect
2	Advanced airway management in pre-hospital care
3	Define time window for time-critical interventions
4	Pre-hospital ultrasound
5	Dispatch/activation criteria for physician-manned EMS
6	Integrated information systems
7	Evaluating quality of care
8	Patient safety in the pre-hospital setting
9	Pre-hospital temperature management in critical care patients
10	Monitoring in the pre-hospital setting
11	Fluid resuscitation in shock
12	Efficient and reliable trauma registries
13	Immobilization techniques
14	Pre-hospital management of stroke
15	Where to go with which patient?
16	Emergency cardiac care in the pre-hospital setting
17	Management of haemorrhagic shock
18	Interhospital transport
19	Does further centralization give better outcomes?
20	Goal-directed therapy studies in pre-hospital critical care
21	EMS systems - regionalization of emergency care
22	Validity and impact of pre-hospital assessment
23	Economic impact of EMS
24	Pre-hospital analgesia, new perspectives
25	Major incident management: How can it be improved?
26	Management of severe head injury
27	Pre-hospital recognition and goal-directed therapy of sepsis
28	Paediatric transport solutions
29	Implementation of new guidelines and research findings
30	Effects of pre-hospital care on quality of life
31	Ethical implications in pre-hospital research
32	Pre-hospital care as a steering system for acute patients
33	Lay person interventions before arrival of EMS
34	Communication and interaction between EMS and hospitals
35	Evaluation of future needs in pre-hospital care
36	Pre-hospital thoracotomy

At the consensus meeting, the expert panel was divided into two groups. Each group was tasked with reaching agreement on three specific questions or subject areas within each research area to focus and prioritise the research efforts. In a final plenary session, the expert panel discussed and made a final decision on the top five areas in addition to three definite research questions, as described above. (Table 2) At the end of the meeting, the expert panel decided to continue the cooperation with a second meeting planned for October 2011.

**Table 2:** The top five priority research areas with key questions to be addressed

Research Area	Key Research Questions to be addressed
Appropriate staffing and training in pre-hospital critical care and the effect on outcomes. This includes the value of physicians in the pre-hospital field.	What staffing and training is required to meet the needs of specific groups of critical care patients in the pre-hospital environment? Is the cost of high-level staffing worthwhile? Which training methods are successful, and how are the skills maintained and assessed?

Advanced airway management in pre-hospital care: what is best for the patient?	What are the indications for advanced airway interventions? What factors influence the decision to intubate, and what is the physician's role in decision-making? When should alternative airway devices or conservative airway manoeuvres be used?

Define time windows for key critical interventions which are indicated in the pre-hospital phase of care.	How does time to definitive in-hospital care influence pre-hospital decisions, and how do pre-hospital decisions influence the time to definitive in-hospital care? Do pre-hospital management protocols result in better adherence to evidence-based guidelines in time-critical conditions? Which clinical situations are time limited or time dependent?

The role of pre-hospital ultrasound.	Which ultrasound examinations can be reliably transferred to the pre-hospital setting? How does pre-hospital ultrasound affect patient management and the patient pathway? How should providers achieve and maintain specific ultrasound skills?

Dispatch/activation criteria for pre-hospital critical care services.	Which criteria accurately identify high acuity patients who require critical care attendance or transport? Do established dispatch systems efficiently target high-resource services? What defines under- and overtriage in specific patient groups, and what rates do current systems produce?

## Discussion

The two most important areas for future research as suggested by the expert panel turned out, not surprisingly, to be two of the most controversial and difficult areas of clinical pre-hospital practice: the role of advanced practitioners in pre-hospital critical care and advanced pre-hospital airway management. Despite having received much attention, both of these areas remain unresolved.

The effect and efficiency of physician manning of pre-hospital EMS has been the subject of several studies in recent years [5,6,44-52]. This subject has been, and remains, controversial and is linked to the debate between advanced and basic life support [52]. The results have been contradictory to some extent, even in similar studies. For example, in two retrospective comparisons of blunt trauma patients treated by physicians or paramedics, Garner et al. [6] found a lower mortality in the physician group, whereas Iirola et al. [45] found a trend towards higher mortality in the physician group. However, both studies found that although treatment in the physician groups was more extensive, it did not delay arrival at the hospital. One review article comparing pre-hospital treatment by physicians or paramedics supported the inclusion of physicians in EMS for pre-hospital trauma care [5], whereas another found increased survival in trauma patients and a trend towards increased survival in cardiac arrest, myocardial infarction and respiratory distress [49]. Both reviews found that the number of articles was few and that the quality of the studies was variable. The topic has remained controversial due to study design and a lack of consistent results in addition to confounding factors such as publication bias in favour of physician-based services, comparisons of different transport methods and differences in interventions performed by the respective groups [5,49].

Pre-hospital airway management is one such advanced intervention. Despite being the topic of several articles, including review articles and a Cochrane review [37,53-55], a recent all-time literature review concluded that the data presented in studies focusing on pre-hospital airway management in adults were deficient and inconsistent [28], making the majority of studies non-conclusive and invalid. In the last two years, several large, well-designed pre-hospital studies have been initiated, and importantly, the first prospective, randomised, controlled clinical trial of pre-hospital intubation in adult patients with severe head injury, was published in 2010 [56]. Nevertheless, while this study represents a milestone in pre-hospital airway management research, it focuses on paramedic intervention, and the authors conclude that more research is needed. Our knowledge of the crucial factors associated with a good outcome of pre-hospital intubation remains poor, and more high-quality studies are needed. In 2009, an Utstein template for documenting and reporting pre-hospital advanced airway management was published [42]. This template may increase the validity of future studies on this subject [57].

The third priority was the timing and necessity of critical interventions. A recurring controversy concerns which advanced interventions should be performed in the pre-hospital setting and which can and should wait until hospital arrival. Established time concepts such as 'the golden hour of trauma' have been challenged due to the lack of scientific evidence [58-60]. Concerning time in pre-hospital critical care, there has been a major debate in recent decades between the main approaches of 'scoop and run' vs. 'stay and play' [29]. Connected to the subject of staffing in the literature, the apparently improved outcome of trauma patients treated by basic life support teams compared with advanced life support teams has been explained partly by the extra time spent on the accident site by the latter, as mentioned in several review articles [27,30,61]. While the focus of the debate has been on treating the patient at the accident scene in contrast to quick transport to definitive care, studies that focus on which procedures can be omitted at what times while considering all variables are scarce. There are several aspects to time questions, such as which conditions/clinical situations really cannot wait, how long it takes to arrive at definitive care in different systems/geographical areas, and the risks vs. benefits of interventions on-scene in particular situations. The questions posed by the group address these issues in specific ways.

Incidentally, time consumption has been one of the major concerns regarding the implementation of the expert panel's priority number four, pre-hospital ultrasound (US) [62]. New technology continues to make in-hospital standards for monitoring and diagnosis applicable in the pre-hospital setting, but the potential benefit to the patient remains to be proven. The expense, time consumption and added weight of taking new technology to scenes can be considerable. The need for education and training to maintain the skills necessary to operate the equipment is also important. The group chose pre-hospital US as the highest-priority technological tool to be evaluated. Small, battery-powered US machines have proven feasible in the pre-hospital field [63,64]. However, despite receiving broad attention in the past decade [65], a review article from October 2010 concluded that there is currently no evidence in the literature to support that pre-hospital US of the abdomen or thorax improves the treatment of trauma patients [66], and a review article of echocardiography in cardiac arrest from June 2010 concluded that no studies so far have shown an improved outcome through the use of this imaging modality [67]. Conversely, a prospective trial concerning ultrasound in cardiac arrest, published in November 2010, found that application of advanced life support-compliant echocardiography in pre-hospital care is feasible, and alters diagnosis and management in a significant number of patients. However, further studies into its effect on patient outcomes were warranted [68]. The clinical situations in which US should be examined as a useful tool are expanding in number, and publications on its use in brain trauma [69], airway management [70], differentiating chronic obstructive pulmonary disease exacerbation from heart failure [71] and rapid treatment of fractures [72] mirror the huge expansion and availability of US in emergency medicine.

The final area of interest to the group is less related to hospital medicine and more to the core business of EMS systems. The group recognised that accurate dispatch is pivotal to success, considering the relatively scarce resources and the importance of early attendance at incidents with time-critical injuries. A systematic review concerning physician-staffed EMS dispatches in 2009 found 34 studies that met the inclusion criteria. However, the study concluded that there are few studies describing the validity of criteria defining an appropriate physician-staffed EMS dispatch and that the results from these studies lack general applicability [9]. A study published in 2010 that provided an overview of dispatch criteria related to physician-staffed EMS organisations in Europe found a lack of uniformity in the use of these criteria for trauma assistance on a national and international level. The study concluded that future research should aim to identify a general set of criteria with the highest discriminating potential. The group recognised that accurate dispatch is pivotal to success, considering the relatively scarce resources and the importance of early attendance at incidents with time-critical injuries.

The recent survey of the 999 EMS Research Forum determined the most important priority topic to be the 'Development of EMS performance measures other than response times for use in performance management, audit and research' [38]. This subject also generated considerable discussion at the consensus meeting, as the value of pre-hospital research is limited in the absence of appropriate outcome measures that reflect the impact of pre-hospital interventions. Current evaluations of the quality of pre-hospital care are frequently as crude as the time from incident to arrival at hospital [73,74]. Pre-hospital interventions that delay transfer time will be difficult to introduce in such systems unless improvements in clinical condition or outcome are measured and demonstrated. When survival to discharge from hospital is used as a measure of pre-hospital care it can be confounded by the entire chain of in-hospital treatment, this makes the effect of pre-hospital interventions difficult to demonstrate. The group discussed alternative measures for evaluating the quality of pre-hospital care, including isolated pre-hospital physiologic parameters and patient satisfaction, as additional outcome measures. The evaluation of the quality of pre-hospital care is of crucial importance to all of the mentioned areas in this exercise, but it did not emerge as a separate research suggestion in the initial rounds. In an article by Jones et al., in 1991, this topic was listed as one of the key questions in pre-hospital research [15]. Twenty years later, the question seems to be as essential as ever.

### Limitations

The scientific value of Delphi Surveys and NGTs has been questioned [75,76]. However, consensus methods continue to be a useful tool for assessing the extent of agreement on matters in which hard evidence is difficult to obtain, and the results can serve as input for other processes [40,77,78]. Some critics of group meetings have argued that verbally skilled participants can monopolise the group with arguments over wording [43] and that 'strong' members of the group may take control of the consensus process to defend their own viewpoints [79]. The project group tried to avoid this problem by anonymising the three first stages and by completing the final ranking prior to the consensus meeting.

The prioritisation system used in our study generated discussion at the meeting as to whether it accurately reflected the expert panel's views. Methods to achieve consensus and methods to prioritise suggestions have been widely discussed, but there is still no method that is considered a gold standard [80]. We gave points to reflect how often a particular subject was suggested in addition to the points obtained by priority. Whether this approach provides a more accurate description of the group's opinions can be questioned; however, this did not affect on the resulting top five areas in this particular case.

Nineteen experts participated in our process. A larger number of researchers from more countries may have improved the scientific value of the project. However, physician-based emergency services continue to be a common supplement to pre-hospital medicine in only a few parts of the world [3,5,6,45,81]. We sought to include countries with well-developed and well-integrated physician-staffed EMS similar to the Scandinavian model. A majority of the delegates (Eleven out of the 19) were from the Scandinavian countries and some major European countries were not present. Invitations were sent to German, French and Czech researchers but unfortunately none of these were able to attend this project. The involvement of clinicians from more countries may have yielded a different priority list, for future projects the group will try to increase the number of countries represented in the expert panel.

## Summary and conclusion

The output of this consensus process has produced a number of clear priorities for the future of research in physician-based pre-hospital critical care. The broad international representation and seniority of the invited experts represents significant combined resources across the European continent. The experts' motivation to initiate studies to address these questions may initiate a new phase in the quality of pre-hospital care research through this process. The expert group hopes that these priorities might work as input for anyone involved in research as they should be of interest not only to the researchers themselves, but also those involved in research financing. By drawing attention to where a group of experts in the field feel that the need for more research is of most urgent importance we hope that grant-awarding bodies will be more inclined to support projects that address these areas. The study applied a modified nominal group technique and reached consensus on the top five priorities for future research in the field: appropriate staffing in pre-hospital critical care, advanced airway management, time windows for critical interventions, pre-hospital ultrasound, and dispatch/activation criteria for pre-hospital physician-manned EMS. It was suggested at the meeting that the entire group should meet again in five years to determine the extent to which the research goals from this consensus process have been fulfilled. Specific projects and cooperation between different countries were discussed during the consensus meeting, and another gathering considering these matters is planned in October 2011.

## Competing interests

The authors declare that they have no competing interests.

## Authors' contributions

EF was the main author and, along with HML, communicated with the members of the expert panel and collected the data. HML conceptualised and initiated the project. HML and DL chaired the consensus meeting, and all authors were involved in the planning of the meeting and the writing process. All authors read and approved the final version of the manuscript.
